# F5. CLOZAPINE RELATED THROMBOCYTOSIS AND THROMBOCYTOPENIA

**DOI:** 10.1093/schbul/sby017.536

**Published:** 2018-04-01

**Authors:** Yuree Kang, Seung-Hyun Shon, Woon Yoon, Jungsun Lee

**Affiliations:** Asan Medical Center

## Abstract

**Background:**

It is well known that clozapine causes hematological side effects such as agranulocytosis, neutropenia, and leukocytosis. But, the results about the effects of clozapine on the number of platelets were not consistent. Although thrombocytopenia or agranulocytosis are regarded as more clinically important side effects, thrombocytosis should be monitored because it may be related with increased the risk of thrombosis and pulmonary embolism. We investigated the effect of clozapine on the number of platelets in schizophrenia patients starting clozapine.

**Methods:**

This was a retrospective chart review study using ABLE, an electrical medical record inquiry system of Asan Medical Center. Among individuals who were diagnosed schizophrenia, who applied clozapine more than three months were included as study subjects. Those who were unable to identify the complete blood count (CBC) at the beginning of the clozapine administration and who did not perform more than one CBC at least every 3 months during the observation period were excluded from the study. CBC scores at baseline and at 1, 3, 6, 9, and 12 months after the initiation of medication were obtained, and the mean platelet counts at the initiation and platelet counts at each observation period were compared by paired t-test. The cumulative incidence of thrombocytopenia (<150000 / mm3) and thrombocytosis (> 450000 / mm3) for one year were also calculated.

**Results:**

Ninety-six patients were enrolled in this study and 50 and 41 subjects were remained at month 6 and 12, retrospectively. There was a significant mean platelet change only at month 1 (275.292 ± 74.464/mL) compared to the initiation of treatment (255.500 ± 74.464/mL) (t=-3.553, p>0.001). The cumulative incidence rates were 3.13% for thrombocytopenia, 6.25% for thrombocytosis.

**Discussion:**

Mandatory hematological monitoring is important in the use of clozapine, and other trajectories as well as the WBC might be of clinical interest.

